# Supplementary material for: Metabolomic Panel for the Diagnosis of Heart Failure with Preserved Ejection Fraction
Source: Int J Mol Sci. 2025 Feb 27;26(5):2102. doi: 10.3390/ijms26052102 (PMC11900465; doi:10.3390/ijms26052102)
Supplement: Supplementary file 1 [file ijms-26-02102-s001.zip › ijms-3421989-supplementary.pdf]

Supplementary

Metabolomic panel for the diagnosis of heart failure with preserved ejection fraction.

Table S1. LC-MS/MS parameters used for AA profiling

Name	Retention time	Precursor ion	Product ion	Cone	CE	Internal Standard
Gly	1.21	76.0	30.0	17	10	13C215N-Gly
13C215N-Gly	1.21	79.0	32.0	17	10	
Ala	1.24	90	44	18	10	D4-Ala
D4-Ala	1.24	94	48	18	10	
Pro	1.37	116.1	70.1	11	13	D4-Ala
Orn	1.08	133.1	70.1	18	20	D6-Orn
D6-Orn	1.08	139.1	76.1	18	20	
Leu	2.94	132.1	86.1	17	10	D3-Leu
D3-Leu	2.91	135.1	89.1	17	10	
ILe	2.68	132.1	86.1	17	10	D3-Leu
Val	1.5	118.1	72.1	19	13	D8-Val
D8-Val	1.5	126.1	80.1	19	13	
Asp	1.26	134	116.1	20	13	D3-Asp
D3-Asp	1.26	137	119.1	20	13	
Glu	1.29	148.1	130	20	13	D5-Glu
D5-Glu	1.29	153	135	20	13	
Met	1.93	150.1	133	18	10	D3-Met
D3-Met	1.93	153.1	136	18	10	
Phe	3.74	166.1	120.1	20	12	D5-Phe
D5-Phe	3.74	171	125	20	12	
Arg	1.11	175.1	70.1	22	21	D7-Arg
D7-Arg	1.11	182	77	22	21	
Cit	1.28	176.1	113.1	16	16	D2-Cit
D2-Cit		178.1	115.1	16	16	
Tyr	2.47	182.1	136.1	20	13	D4-Tyr
D4-Tyr		186	140	20	13	
Ser	1.22	106	60	20	13	D4-Ala
Thr	1.28	120	56	20	13	D5-Phe
Lys	1.08	147	84	20	13	D4-Ala
Trp	4.23	205	188	20	13	D5-Phe
His	1.10	156	110	20	13	D4-Ala

Table S2. LC-MS/MS parameters used for AC profiling

Analyte	Abbreviation	Precursor ion	Product ion	CE
Carnitine	C0	162	103	17
Carnitine-D9	C0-ISTD	171	103	17
Acetylcarnitine	C2	204	85	18
Acetylcarnitine-D3	C2-ISTD	207	85	18
Propionylcarnitine	C3	218	85	19
Propionylcarnitine -D3	C3-ISTD	221	85	19
Butyrylcarnitine	C4	232	85	20
Butyrylcarnitine-D3	C4-ISTD	235	85	20
Valerylcarnitine	C5	246	85	20
Valerylcarnitine-D9	C5-ISTD	255	85	20
Tiglylcarnitine	C5:1	244	85	20
	C5-DC	276	85	20
Hexanoylcarnitine	C6	260	85	22
Hexanoylcarnitine-D3	C6-ISTD	263	85	22
Octanoylcarnitine	C8	288	85	24
Octanoylcarnitine -D3	C8-ISTD	291	85	24
Octenoylcarnitine	C8:1	286	85	24
Decanoylcarnitine	C10	316	85	26
Decanoylcarnitine -D3	C10-ISTD	319	85	26
Decenoylcarnitine	C10:1	314	85	26
Decadienylcarnitine	C10:2	312	85	26
Dodecanoylcarnitine	C12	344	85	27
Dodecanoylcarnitine -D3	C12-ISTD	347	85	27
Dodecenoylcarnitine	C12:1	342	85	27
Tetradecadienylcarnitine	C14:2	368	85	27
Hexadecanoylcarnitine	C16	400	85	28
Hexadecanoylcarnitine -D3	C16-ISTD	403	85	28

Octadecanoylcarnitine	C18	428	85	28
Octadecanoylcarnitine - D3	C18-ISTD	431	85	28
Octadecenoylcarnitine	C18:1	426	85	26
Octadecadienylcarnitine	C18:2	424	85	20
Hydroxyvalerylcarnitine	C5-OH	262	85	26
Hydroxyhexadecenoylcarnitine	C16:1-OH	398	85	26
Hydroxyoctadecenoylcarnitine	C18:1-OH	442	85	28

Table S3. LC-MS/MS parameters used for tryptophan metabolism profiling

Analyte	RT, min	Registered ions		Fragmentor	CE
		Precursor ion	Product ion		
Kynurenine	4,6	209,1	146	95	30
Kynurenine	4,6	209,1	94	95	10
Antranillic	6,5	138,1	120,1	95	10
Antranillic	6,5	138,1	92,0	95	20
Kynurenic acid	6,6	190,1	144,1	95	20
Dopamin	3.2	154.1	137	90	10
Dopamin	3.2	154.1	119.1	90	20
Dopamin	3.2	154.1	91	90	30
Serotonin	5.6	160.1	132.1	120	20
Serotonin	5.6	160.1	105.1	120	30
Neopterin	1.5	254.1	206.1	100	20
Neopterin	1.5	254.1	190.1	100	20
Biopterin	2.3	238.1	194	90	20
Biopterin	2.3	238.1	178	90	20
Tryptophan	6.4	205.1	146.1	100	20
Hiaa	6.1	192.1	146.1	100	20
Indol-3-butyric	7.76	204.2	144.2	100	25
Indol-3-butyric	7.76	204.2	130.1	100	25
3-indolepropionic	7.5	190,1	130,1	95	20
Indol-acrilyc	7.4	188.1	115.1	100	25
Indol-acetic	7.2	176,1	130,1	95	20
Indole-3-carboxaldehyde	7.3	146,1	118,1	95	15
Indole-3-carboxaldehyde	7.3	146,1	91,1	95	30
Tryptamine	8	161,1	144,1	95	10
Tryptamine	8	161,1	115.1	95	30
Dl-indole-3-lactic acid	6.8	206,1	118,1	95	22
Quinolinic acid	1.8	168,1	106.1	95	15
Quinolinic acid	1.8	168,1	78.1	95	30
Xanturenic acid	6.6	206	160	95	20
Xanturenic acid	6.6	206	160	95	30
Choline		104	60	90	20
6-hydroxy nicotinic acid	3.8	140	122	100	15
2-hydroxytryptophan		221	162	90	20

Table S4. LC-MS/MS parameters used for ADMA and SDMA profiling

Name	Retention time	Precursor ion	Product ion	Cone	CE	Internal Standard
Arg		175.1	70.1	22	21	D7-Arg
D7-Arg		76.0	30.0	22	21	
ADMA		203.2	46.1	22	21	D7-Arg
SDMA		203.2	172.1	22	21	D7-Arg
Choline		60.1	104.1	22	21	D7-Arg
